# PRIMARY INTRACRANIAL TUMORS: PROGNOSTIC FACTORS OF ORAL INTAKE SCALE OF PATIENTS PRE AND POST SURGICAL TREATMENT

**DOI:** 10.1590/S0004-2803.24612026-022

**Published:** 2026-09-28

**Authors:** Luisa Vargas BRAZ, Ana Maria FURKIM, Diego Fernando Dorneles BILHERI, Vinicius Alexandre ALIANO, Fernanda Yasmin Odila Maestri Miguel PADILHA

**Affiliations:** 1 Universidade Federal de Santa Catarina, Mestranda do Programa de Pós-Graduação em Fonoaudiologia na Atenção à Saúde de Média e Alta Complexidade, Florianópolis, SC, Brasil.; 2 Universidade Federal de Santa Catarina, Professora do Curso de Graduação e do Programa de Pós Graduação em Fonoaudiologia, Florianópolis, SC, Brasil.; 3 Universidade Federal de Santa Catarina, Laboratório de Pesquisa Ensino e Extensão em Disfagia, Florianópolis, SC, Brasil.; 4 Hospital Governador Celso Ramos, Serviço de Fonoaudiologia, Florianópolis, SC, Brasil.; 5 Hospital Governador Celso Ramos, Programa de Residência Médica em Neurocirurgia, Florianópolis, SC, Brasil.; 6 Hospital Baia Sul, Serviço de Fonoaudiologia do Grupo Baia Sul, Florianópolis, SC, Brasil.

**Keywords:** Brain neoplasms, deglutition disorders, eating, speech, language and hearing sciences, Neoplasias encefálicas, transtornos de deglutição, ingestão de alimentos, fonoaudiologia

## Abstract

**Background:**

Primary intracranial tumors are rare neoplasms associated with high morbidity and mortality, whose location, size, and impact on neurological and cognitive status influence treatment and survival.

**Objectives:**

To characterize prognostic factors using the Functional Oral Intake Scale in patients diagnosed with primary intracranial tumors undergoing surgical treatment and hospitalized in a reference hospital in the state of Santa Catarina, Brazil.

**Methods:**

This is a cross-sectional, retrospective, observational study with secondary data collection and analysis of electronic medical records. Medical records of patients admitted to Neurosurgery and undergoing surgical treatment between January 2021 and March 2023 were selected. Based on the information obtained, a correlation was established between the aforementioned scale and age, sex, tumor topography, and classification.

**Results:**

The 68 patients analyzed were a heterogeneous group, showing tumors in various brain locations (left hemisphere, right hemisphere, central regions, and posterior fossa). A higher prevalence was observed in women. The most prevalent tumor was meningioma, with the left cerebral hemisphere being the most affected. Regarding the Functional Oral Intake Scale, the group of patients with posterior fossa tumors showed greater variation when comparing the level at admission to the patient’s immediate postoperative level. Two patients presented Level 1 with the use of an alternative feeding route in the immediate postoperative period; these patients were followed up by the speech therapy team, progressing to Levels 5 and 6 at hospital discharge.

**Conclusion:**

It was observed that the highest prevalence of decreased/restricted oral food intake occurred in patients with primary intracranial tumors of the posterior fossa. No other prognostic factors for decreased/restricted oral intake were found in the sample studied, besides tumor location.

## HIGHLIGHTS

• Evaluated prognostic factors for oral intake using FOIS in neurosurgical patients with primary intracranial tumors.

• Conducted a retrospective cross-sectional study of 68 medical records (2021-2023), correlating FOIS with age, sex, tumor topography, and classification.

• Posterior fossa tumors showed greater postoperative FOIS decline; two patients required alternative feeding (FOIS 1) but progressed to Levels 5-6 at discharge.

• Tumor location, particularly posterior fossa, was the only factor associated with decreased or restricted oral intake in the sample studied.

## RESUMO

**Contexto:**

Os tumores intracranianos primários são neoplasias raras associadas a alta morbidade e mortalidade, cuja localização, tamanho e impacto no estado neurológico e cognitivo influenciam o tratamento e a sobrevida.

**Objetivo:**

Caracterizar os fatores prognósticos pela escala do Nível de Ingestão por Via Oral em pacientes com diagnóstico de tumores intracranianos primários submetidos ao tratamento cirúrgico internados em um hospital referência do estado de Santa Catarina, Brasil.

**Métodos:**

Trata-se de estudo transversal, retrospectivo, observacional com coleta de dados secundários e análise de prontuários eletrônicos. Foram selecionados prontuários de pacientes internados pela Neurocirurgia submetidos a tratamento cirúrgico, no período de janeiro de 2021 até março de 2023. De acordo com as informações obtidas, foi realizada a relação entre a referida escala com a idade, sexo, topografia e classificação dos tumores.

**Resultados:**

Os 68 pacientes analisados foram um grupo heterogêneo, mostrando tumores em várias localizações cerebrais (hemisfério esquerdo, direito, regiões centrais e fossa posterior). Observou-se prevalência maior em mulheres. O tumor mais prevalente foi o meningioma, sendo o hemisfério cerebral esquerdo o mais acometido. Em relação à Escala do Nível de Ingestão por Via Oral, o grupo de pacientes com tumores de fossa posterior apresentou maior variação, quando comparado o nível de entrada na internação com o nível pós-cirúrgico imediato do paciente. Dois pacientes apresentaram Nível 1 com uso de via alternativa de alimentação no pós operatório imediato, os mesmos fizeram acompanhamento com a equipe de fonoaudiologia, evoluindo para Nível 5 e 6 na alta hospitalar.

**Conclusão:**

Observou-se que a maior prevalência de diminuição/restrição de alimentos ingeridos por via oral ocorreu em pacientes com tumores intracranianos primários de fossa posterior. Não foram encontrados outros fatores prognósticos de diminuição/restrição de alimentação por via oral na amostra estudada, além da localização do tumor.

## INTRODUCTION

Primary intracranial tumors of the brain and meningeal structures, collectively called primary brain tumors, are a group of neoplasms originating from supporting cells of nervous tissue. They are rare, with a five-year overall survival rate of 33.4%, corresponding to 2% of all known cancers, and despite their low incidence, they have high morbidity and mortality rates^1^ ^,^ ^2^. The main neurological symptoms presented by patients with intracranial tumors are: paralysis, visual disturbance, sensory loss, altered consciousness, memory loss, and functional impairment, including dysphagia. Therefore, the location and size of the tumor, as well as the patient’s neurological and cognitive status, influence the type of treatment and the patient’s chances of survival^3^ ^,^ ^4^_._

Surgical intervention is the most common treatment for primary intracranial tumors and the tumor resection should be maximal, provided that neurological function is not compromised, as this is directly related to the prognosis of the disease. Neurosurgery is the most feared treatment by patients and their families, considering that the brain regulates important bodily functions such as breathing, motor function, behavior, and circulation^4^ ^,^ ^2^.

Depending on its location, an intracranial tumor can interfere with the swallowing biomechanics, considering that the control of this process involves an integrated network of structures in the cerebral cortex and brainstem of the Central Nervous System (CNS)^5^. Swallowing is a vital physiological function, responsible for transporting food from the mouth to the stomach through a complex reflex of the stomatognathic system in conjunction with the CNS^6^. Normal swallowing is characterized by physiological behaviors that result in the safe and efficient movement of food, liquids, or other substances to the stomach. However, difficulty (motor and/or sensory) in one or more anatomical or physiological components of the oral, pharyngeal, or esophageal stages of swallowing and/or the gastroesophageal tract, resulting from neurological and/or structural alterations, is defined as dysphagia^7^ ^,^ ^8^ ^,^ ^5^. The cranial nerves V (trigeminal), VII (facial), IX (glossopharyngeal), X (vagus), XI (accessory) and XII (hypoglossal) are responsible for the motor and sensory control of each phase of swallowing^7^.

The consequences of dysphagia include: malnutrition, dehydration, aspiration pneumonia, compromised overall health, chronic lung diseases and asphyxia, which can lead to death^8^. Therefore, it is the responsibility of the multidisciplinary team to promote and act on the clinical stability of the dysphagic patient, establishing, when possible, safe oral feeding (OF), reducing the time of use of alternative feeding tubes and the length of hospital stay^9^. The functional level of food and liquid oral intake in dysphagic patients is typically established after clinical examination and, for this purpose, the Functional Oral Intake Scale (FOIS)^10^ can be used as a marker to document clinical changes and evolutions after speech-language pathology assessment. FOIS consists of a classification for the evolution of the level of oral intake, ranging from level one (no oral intake) to level seven (total oral intake without restrictions). 

Given the topic discussed, the objective of this study was to characterize the prognostic factors of oral intake levels in patients hospitalized in a reference Neurology hospital in the state of Santa Catarina with a diagnosis, in their medical records, of primary intracranial tumors who underwent surgical treatment.

## METHODS

This is a cross-sectional, retrospective, observational study with analysis of electronic medical records and collection of secondary data. Medical records of patients admitted to the Neurosurgery department who underwent neurosurgical treatment for primary intracranial tumors at a reference Neurology hospital in the state of Santa Catarina, located in the city of Florianópolis, were analyzed for the period from January 2021 to March 2023.

Patients were located using surgical procedure codes available in the hospital health management system - Sistema de Gestão de Saúde Hospitalar (SGS)*.* The data were obtained from electronic medical records, based on medical and multidisciplinary progress notes, including sociodemographic information (age, sex, ethnicity, marital status, and education level) and clinical information (medical history, type of brain tumor, topographic diagnosis and length of hospital stay). To analyze the data regarding the level of oral intake, the Functional Oral Intake Scale^10^ (Table 1) was used, according to the medical prescription and/or speech therapy assessment of the patient at four points during hospitalization: date of admission, pre-surgery, post-surgery and at the time of hospital discharge.

**TABLE 1:** Functional Oral Intake Scale - FOIS^10^.

FOIS level	Comment
Level 1	Nothing by mouth.
Level 2	Tube dependent with minimal attempts of food or liquid.
Level 3	Tube dependent with consistent oral intake of food or liquid.
Level 4	Total oral diet of a single consistency.
Level 5	Total oral diet with multiple consistencies, but requiring special preparation or compensations.
Level 6	Total oral diet with multiple consistencies without special preparation, but with specific food limitations.
Level 7	Total oral diet with no restrictions.

From the data collection, of the 220 medical records, patients were excluded according to the criteria described in the flowchart (Figure 1): patients aged 60 years or older (62 individuals); patients diagnosed with secondary brain tumors (18 individuals); patients with previous neurological impairments or tumor recurrence (28 individuals); patients with Chronic Obstructive Pulmonary Disease (COPD) and/or other respiratory diseases (0 individuals); patients with esophageal diseases (1 individual); patients who presented a decreased level of consciousness in the pre- and/or postoperative period (Glasgow Coma Scale - GCS <8) (5 individuals); patients who underwent tracheostomy (0 individuals); patients with spinal cord neoplasms (1 individual); patients who did not have intracranial tumors or whose diagnosis was inconclusive (5 individuals); patients whose medical records did not contain all data regarding the variables analyzed (20 individuals); deaths (11 individuals); duplicates (3 individuals).

**FIGURE 1:**
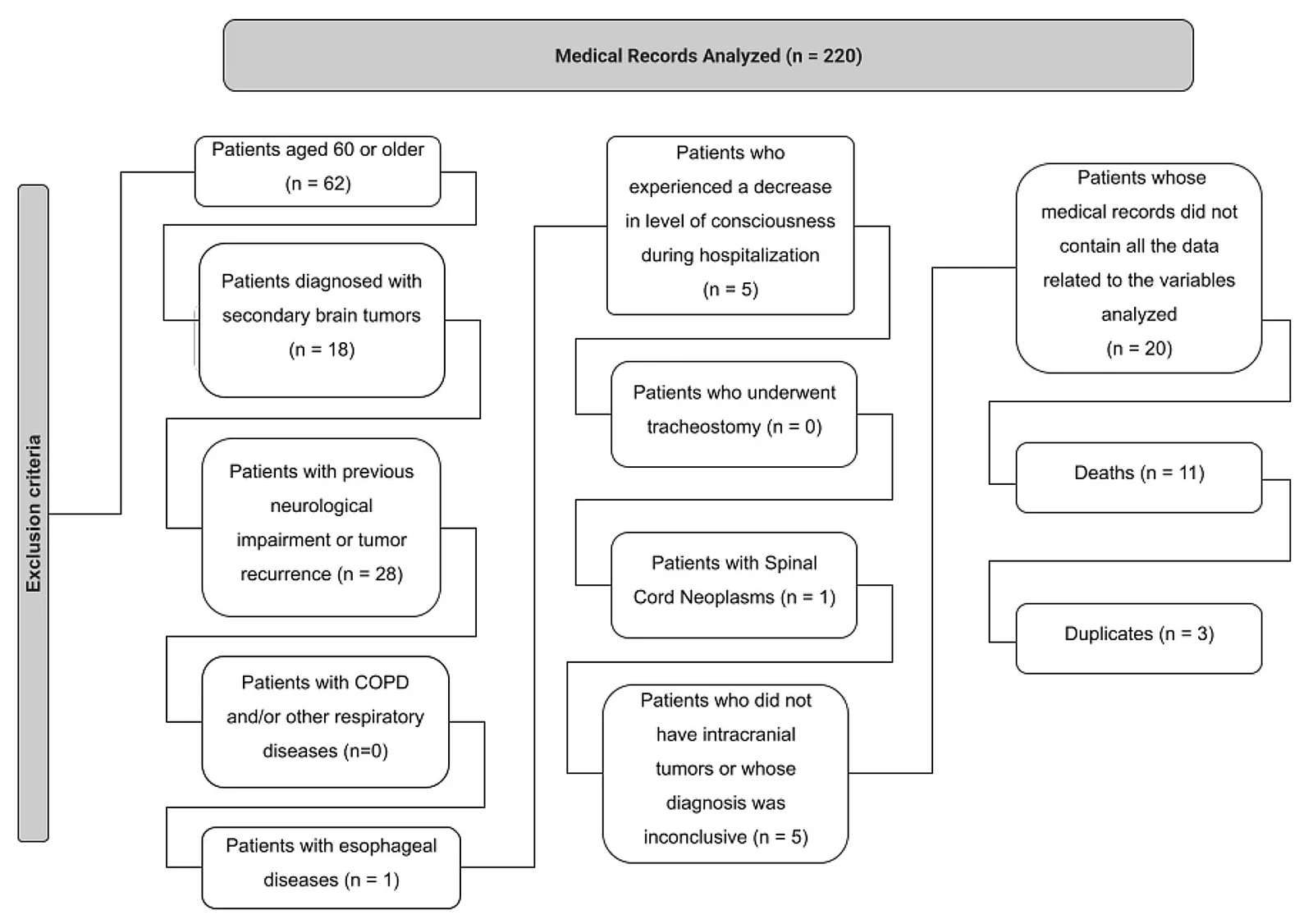
Flowchart of exclusion criteria.

This study was approved by the institution’s Research Ethics Committee involving Human Subjects under number 68044723.0.0000.5360. The present study is exempt from the Informed Consent Form considering it is an observational, descriptive, and retrospective study that will use only information from medical records and the institutional information systems mentioned above to obtain clinical data available at the institution, with no plan to use biological material. All data were handled and analyzed anonymously, without nominal identification of the research participants. The results arising from the study will be presented in aggregate form, not allowing the individual identification of participants.

All the data mentioned above were organized in an Excel® spreadsheet (Microsoft Corporation), and for statistical and descriptive analysis, mean values and percentage analyses were calculated, as well as the relationship among the variables analyzed.

According to the information obtained from medical records using the FOIS, the relationship among age, sex, tumor topography, and tumor classification was characterized. Tumor topography was divided into tumors of the right hemisphere, left hemisphere, posterior fossa tumors, and tumors in central regions. In the latter group, for better data analysis, intraventricular tumors, tumors of the sella turcica, and tumors of the pituitary gland or pineal gland were grouped together.

## RESULTS

The 68 patients analyzed were a heterogeneous group, showing tumors in various brain locations, including the left and right hemispheres, central regions, and posterior fossa.

Based on sociodemographic and health data (Table 2), a slightly higher prevalence was observed in women (52.94%) compared to men (47.06%), with a mean age of 40.53 years (SD ± 11.82). The highest concentration of diagnoses occurred in the 50-59 age group (29.4%).

**TABLE 2:** Table of sociodemographic and health characteristics.

Sociodemographic characteristics	n	n
**Age Range**		
18-29 years	16	23,53
30-39 years	16	23,53
40-49 years	16	23,53
50-59 years	20	29,41
**Sex**		
Man	32	47,06
Woman	36	52,94
**Ethnicity**		
White	62	91,18
Brown	5	7,35
Black	1	1,47
**Marital Status**		
Married	39	57,35
Single	24	35,29
Divorced	2	2,94
Widowed	1	1,47
No data in medical records	2	2,94
**Education level**		
Illiterate to Ementary School	23	33,82
High School	29	42,65
Higher Education	13	19,12
No data	12	17,65
Health Characteristics		
**Hypertension**	n	%
Yes	16	23,53
**Diabetes Mellitus**		
Yes	7	10,29
Smoker		
Yes	6	8,82
**Alcohol Use**		
Yes	1	1,47
**Others comorbidities**	21	30,88

Results expressed through frequency analysis.

Regarding the most prevalent tumors types in the sample (Figure 2), Meningioma (23.5%), Glioblastoma (16.1%) and Vestibular Schwannoma (13.2%) were identified, with Meningiomas and Vestibular Schwannomas being more prevalent in the female population (75% and 66.6%) and Glioblastomas in the male population (83.3%).

**FIGURE 2:**
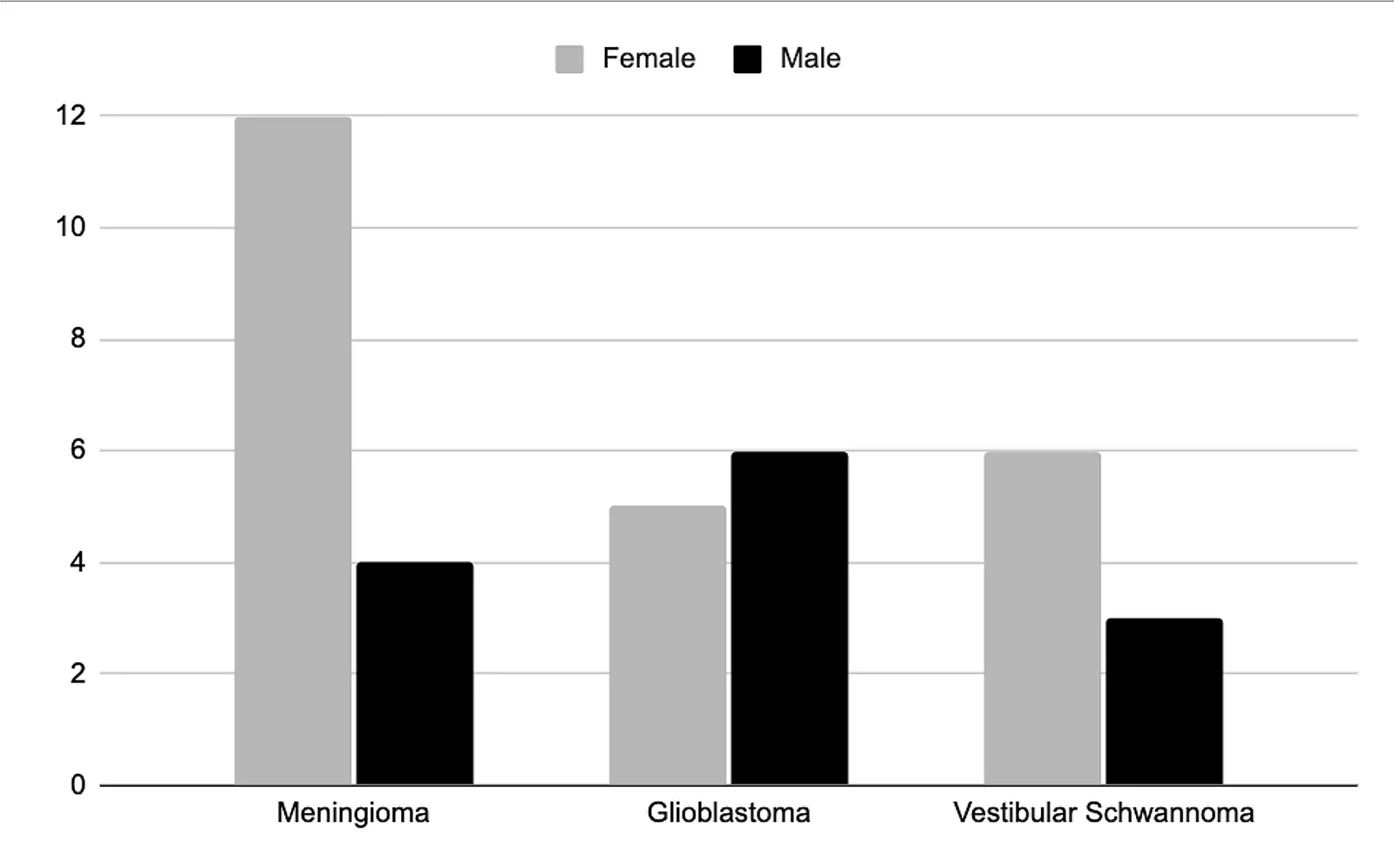
Prevalent tumors in the sample in relation to sex.

Regarding the topography (Table 3), a discrepancy was found in relation to sex, as the left hemisphere predominates in women and the right in men. However, in the total group studied, it was observed that the left cerebral hemisphere was the most affected (30,88%), followed by the posterior fossa and the right hemisphere with 18 individuals in each region (26,47%). The speech-therapy team treated 13 patients with posterior fossa tumors, 4 with tumors in central regions, 2 with tumors in the left hemisphere, and 5 with tumors in the right hemisphere.

**TABLE 3:** Tumor topography in relation to sex.

Topography	Female (n = 36)		Male (n = 32)		Total (n = 68)	
	n	%	n	%	n	%
Central	6	16,66	5	15,625	11	16,17
Right Hemisphere	7	19,44	11	34,375	18	26,47
Left Hemisphere	14	38,88	7	21,875	21	30,88
Posterior Fossa	9	25	9	28,125	18	26,47

Results expressed through frequency analysis.

Considering the FOIS, among the 68 patients analyzed, it was observed, according to Table 4, that the group of patients with posterior fossa tumors showed greater variation in the FOIS scale when comparing the admission score (16 patients with FOIS 7 and 2 with FOIS 5) with the postoperative score (which ranged between FOIS 7, 6, 5, 4, and 1).

**TABLE 4:** Relationship between FOIS during hospitalization and tumor topography.

Topography	Admission			Pre-surgery			Post-surgery			HD		
	n (FOIS)	%	STsST	n (FOIS)	%	ST	n (FOIS)	%	ST	n (FOIS)	%	ST
Posterior												
Fossa												
(n=18)	16 (7)	88,89	-	16 (7)	88,89	-	1 (7)	5,56	-	5 (7)	27,78	3
	2 (5)	11,11	2	1 (6)	5,56	1	4 (6)	22,22	2	7 (6)	38,89	6
				1 (5)	5,56	1	9 (5)	50,00	8	6 (5)	33,33	4
							1 (4)	5,56	-			
							3 (1)	16,67	3			
Central												
(n=11)	8 (7)	72,73	-	8 (7)	72,73	-	2 (7)	18,18	-	6 (7)	54,55	1
	2 (5)	18,18	1	1 (6)	9,09	1	1 (6)	9,09	-	1 (6)	9,09	1
	1 (1)	9,09	-	1 (5)	9,09	-	8 (5)	72,73	4	4 (5)	36,36	2
				1 (1)	9,09	-						
Right												
(n=18)	14 (7)	77,78	-	18 (7)	100	-	2 (7)	11,11	-	10 (7)	55,56	-
	3 (6)	16,67	-				4 (6)	22,22	-	4 (6)	22,22	3
	1 (1)	5,56	-				12 (5)	66,67	5	4 (5)	22,22	2
Left												
(n=21)	21 (7)	100,00	-	21 (7)	100	-	7 (7)	33,33	-	13 (7)	61,90	-
			-				4 (6)	33,33	-	3 (6)	14,29	-
			-				10 (5)	47,62	2	5 (5)	23,81	2

FOIS: functional oral intake scale. HD: hospital discharge. ST: speech therapy services results expressed through frequency analysis.

Analyzing only the patients with posterior fossa tumors (Table 4), three patients presented FOIS 1 with the use of an enteral nutrition (EN) via feeding tube in the immediate postoperative period. These patients received follow-up from the speech-language pathology team and progressed to FOIS 5 and 6 at hospital discharge, without the need for EN. Only one patient presented FOIS 1 (central tumor) with nothing by mouth (NPO) at admission and in the preoperative period; however, this patient did not use an EN and remained in NPO only for the procedure.

None of the patients in the sample presented with FOIS 3 or lower at hospital discharge, meaning that no patient required EN after hospital discharge. Regarding the overall sample, 59 presented with FOIS 7 at admission; of these, only 12 maintained FOIS 7 in the immediate postoperative period, and despite this difference, 44 patients progressed to FOIS 7 by hospital discharge.

Observing the relationship between FOIS at hospital discharge (HD) and the topography of intracranial tumors (Table 5), it was found that at HD 34 patients presented FOIS 7 (50%) and 34 presented FOIS 6 or less (50%). Of these, 13 patients (38,34%) had posterior fossa tumor topography. It was possible to observe that patients with posterior fossa tumors showed greater variation in the FOIS in the postoperative period and at HD compared with the other regions analyzed.

**TABLE 5:** Relationship between hospital discharge index (HD) and intracranial tumor topography.

Topography	FOIS 7 (n=34)		FOIS ≠ 7 (n=34)	
	n	%	n	%
Central	6	17,65	5	14,71
Right Hemisphere	10	29,41	8	23,53
Left Hemisphere	13	38,24	8	23,53
Posterior Fossa	5	14,71	13	38,24

Results expressed through frequency analysis.

## DISCUSSION

It was observed in this study that the highest prevalence of decreased/restricted oral intake occurred in patients with primary intracranial tumors of the posterior fossa. Furthermore, no other prognostic factors for decreased/restricted oral intake were identified, apart from tumor location.

According to the Instituto Nacional de Câncer^11^, the incidence of CNS cancer in 2023 was estimated at 11,74 cases per 100,000 men and 8,16 cases per 100,000 women in Santa Catarina, Brazil. The clinical manifestations of intracranial tumors generally depend on the anatomical region of the brain or adjacent structures involved, as well as tumor size and the patient’s neurological and cognitive status, factors that also influence the type of treatment and the patient’s chances of survival^4^ ^,^ ^12^.

In the present study, a slightly higher prevalence of primary intracranial tumors was observed in women (52,54%). The female predominance identified is consistent with the findings of studies by Mendes et al.^13^ and Rodrigues et al.^17^, which reported 67% and 65% of their samples as female, respectively. However, this contrasts with 2023 data on central nervous system neoplasms, which show a higher incidence in the male population^11^.

Regarding classification, the most prevalent primary intracranial tumors are gliomas and meningiomas^15^ ^,^ ^16^. The literature identifies glioblastoma as the most common malignant brain tumor (14,3% of all tumors and 49,1% of malignant tumors), while meningioma is classified as the most common nonmalignant tumor (39% of all tumors and 54,5% of nonmalignant tumors)^17^. In this study, there was a predominance of patients with meningioma (23,5%), followed by glioblastoma (16,1%). Similar findings have been reported in other studies about these populations^13^ ^,^ ^14^. Quantitatively, the studies showed differences in prevalence percentages, possibly due to sample size, as also observed in the present study.

Furthermore, when comparing by sex, it was observed that meningiomas were more prevalent in the female population (75%) and glioblastomas in the male population (83,3%), a finding also confirmed by the literature^14^ ^,^ ^16^ ^,^ ^17^. In general, the literature shows that brain tumors are more prevalent in women, mainly meningiomas and pituitary tumors. However, considering divisions based on cellular origin, the literature indicates that gliomas, embryonal tumors, and germ cell tumors tend to occur more frequently in men^17^ ^,^ ^18^ ^,^ ^19^.

Topographically, the left cerebral hemisphere was the most affected (30,88%) in this study, followed by the posterior fossa region and the right hemisphere, each accounting for 26,47% of the individuals. This finding differs from the study conducted by Buffon et al.^12^, which, in an analysis of 401 patients, observed a prevalence of tumors in the right hemisphere of 42,4%. In contrast, another study presents similarities with the present article, in which, among 106 cases, 39% of tumors were supratentorial and 11% infratentorial^13^. In addition, the literature reports a higher prevalence of tumors in the frontal lobe, followed by tumors in the temporal lobe^12^ ^,^ ^17^; however, these variables were not considered in this study.

Based on the results obtained, when correlating tumor topographies with FOIS during hospitalization, a greater variation in the FOIS scale was observed in patients with posterior fossa tumors, considering both the admission and postoperative FOIS scores. This finding can be confirmed by considering that tumors in the pons and cerebellum, tumors that compress regions of the lower cranial nerves, and even cortical tumors that affect motor programming, anticipation, and cognition, can be aggressive to the oral/pharyngeal phase of swallowing.

Furthermore, the literature describes central control of swallowing as occurring through an organization of neural elements in the brain and brainstem. In general, the CNS initiates and coordinates the muscles involved in the phases of swallowing by activating cortical and subcortical structures of supratentorial and infratentorial origin, especially the cranial nerves involved in swallowing, which provide motor and sensory control of each phase of swallowing, as well as the cerebellum, spinal cord, and some spinal nerves^5^ ^,^ ^7^.

A study conducted by Lapa et al.^20^, specifically in an adult population with posterior fossa tumors, analyzed a sample of 26 patients, of whom 60% were identified as having dysphagia in the preoperative period. The authors did not observe an increase in the number of dysphagia cases after surgery; however, there was a worsening of preexisting swallowing function in about half of the subjects to the point of requiring an enteral nutrition. Therefore, tumors located at the posterior skull base are directly and anatomically related to nerves that may cause swallowing disorders due to paralysis of the pharyngeal musculature^21^. These structures also play a fundamental role in the biomechanics of swallowing, such that dysphagia may occur as a clinical manifestation of the tumor itself or as a possible sequela after surgical removal^20^.

In the literature, few studies were found correlating swallowing disorders with intracranial tumors, with research predominantly involving pediatric populations. The relationship most frequently addressed in the literature concerns dysphagia in cases of posterior fossa tumors, both in pediatric and adult populations^20^ ^-^ ^23^. Therefore, it is important to conduct further research on this topic in other brain regions in the adult population, as well as to consider mandatory swallowing assessment in patients with posterior fossa tumors.

### Study limitations

Speech-language pathology follow-up in this population occurred only upon medical request; therefore, many patients did not receive a speech-language pathology evaluation regarding swallowing. This aspect may have influenced the results, since dysphagia may have been underdiagnosed or overlooked due to the lack of specialist assessment. Furthermore, it was not possible to quantify the progression of patients who were not treated by the speech-language pathology team, considering the possibility that the lack of improvement in these patients may have occurred due to the absence of care from the team.

The results are restricted to the studied population, from which patients with previous risk factors for dysphagia were excluded. Therefore, clinicians should be aware of possible variation in these data when managing patients at risk for dysphagia (older adults, patients with secondary brain tumors, patients with previous neurological impairments or tumor recurrence, patients with COPD and/or other respiratory diseases, patients with esophageal diseases, patients who presented decreased level of consciousness during hospitalization, and patients who underwent tracheostomy).

## CONCLUSION

In this study, it was observed that the highest prevalence of decreased/restricted oral intake occurred in patients with primary intracranial tumors of the posterior fossa. No other prognostic factors for decreased/restricted oral feeding were found in the sample studied, other than tumor location.

It is emphasized that the findings represent an observational association in a highly selected population and cannot be generalized to all patients with primary intracranial tumors or used solely for clinical prognostic stratification. Therefore, further studies should be conducted with a prospective design, larger sample size, and objective assessment of swallowing.

## Data Availability

Data available upon request
